# Supplementary material for: New 12,23-Epoxydammarane Type Saponins Obtained from Panax notoginseng Leaves and Their Anti-Inflammatory Activity
Source: Molecules. 2020 Aug 20;25(17):3784. doi: 10.3390/molecules25173784 (PMC7504330; doi:10.3390/molecules25173784)
Supplement: Supplementary file 1 [file molecules-25-03784-s001.pdf]

Supporting Information

New 12,23-Epoxydammarane Type Saponins Obtained from *Panax notoginseng* Leaves and Their Anti-Inflammatory Activity

JingyaRuan[1], Ying Zhang [2], Wei Zhao [1], Fan Sun [1], Lifeng Han [1], Haiyang Yu [2], Lijie Wu [1], Yi Zhang [1,2,*] and Tao Wang [1,2,*]

[1] Tianjin Key Laboratory of TCM Chemistry and Analysis, Tianjin University of Traditional Chinese Medicine, 10 Poyanghu Road, West Area, Tuanbo New Town, Jinghai District, 301617, Tianjin, China; Ruanjy19930919@163.com (J.R.); sf18435165322@163.com (F.S.); zhaowei126123@126.com (W.Z.); hanlifeng_1@sohu.com (L.H.); wulj0816@163.com (L.W.)

[2] Institute of TCM, Tianjin University of Traditional Chinese Medicine, 10 Poyanghu Road, West Area, Tuanbo New Town, Jinghai District, 301617, Tianjin, China; zyingtzy@163.com (Y.Z.); hyyu@tjutcm.edu.cn (H.Y.)

* Correspondence:zhwwxzh@tjutcm.edu.cn (Y.Z.); wangtao@tjutcm.edu.cn (T.W.); Tel./Fax: +86-22-5959-6168 (T.W.)

Figure S1 ^{1}H NMR (500 MHz, C_5D_5N) spectrum of **1** 3
Figure S2 ^{13}C NMR (125 MHz, C_5D_5N) spectrum of **1** 3
Figure S3 ^{1}H ^{1}H COSY (C_5D_5N) spectrum of **1** 4
Figure S4 HSQC (C_5D_5N) spectrum of **1** 4
Figure S5 HMBC (C_5D_5N) spectrum of **1** 5
Figure S6 HSQC-TOCSY (C_5D_5N) spectrum of **1** 5
Figure S7 ESI-Q-Orbitrap-MS spectrum of **1** 6
Figure S8 ^{1}H NMR (600 MHz, C_5D_5N) spectrum of **2** 7
Figure S9 ^{13}C NMR (150 MHz, C_5D_5N) spectrum of **2** 7
Figure S10 ^{1}H ^{1}H COSY (C_5D_5N) spectrum of **2** 8
Figure S11 HSQC (C_5D_5N) spectrum of **2** 8
Figure S12 HMBC (C_5D_5N) spectrum of **2** 9
Figure S13 NOSEY (C_5D_5N) spectrum of **2** 9
Figure S14 ESI-Q-Orbitrap-MS spectrum of **2**. 10
Figure S15 MTT assay of compounds **1** and **2** at the concentration of 25 μM on RAW 264.7 cells 11
Figure S16 Raw quantification data for figure 3.................. 12
Figure S17 Raw quantification data for figure 4.................. 13

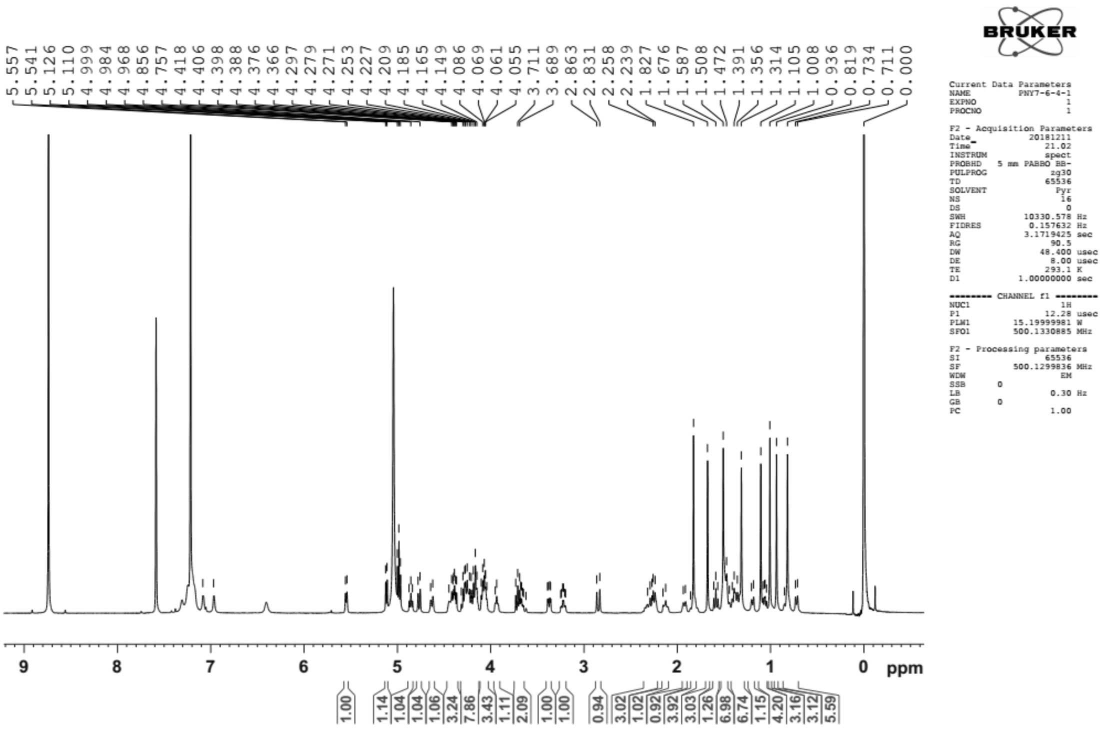

Figure S1 ^{1}H NMR (500 MHz, C_5D_5N) spectrum of **1**

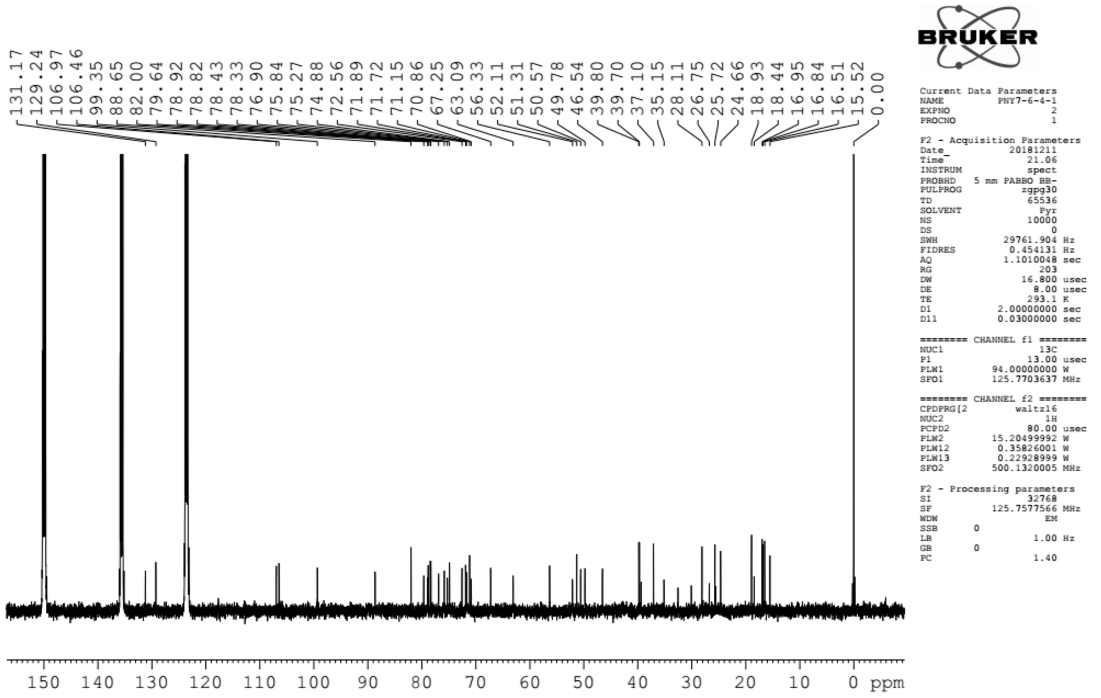

Figure S2 ^{13}C NMR (125 MHz, C_5D_5N) spectrum of **1**

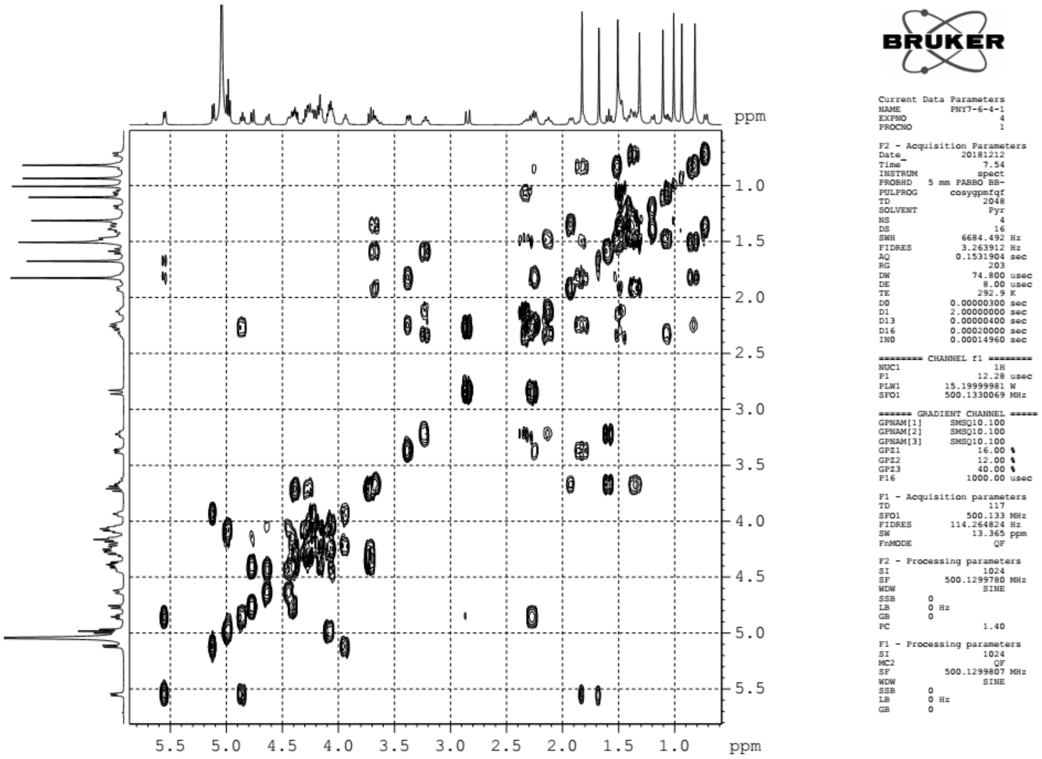

Figure S3 ^{1}H ^{1}H COSY (C_5D_5N) spectrum of **1**

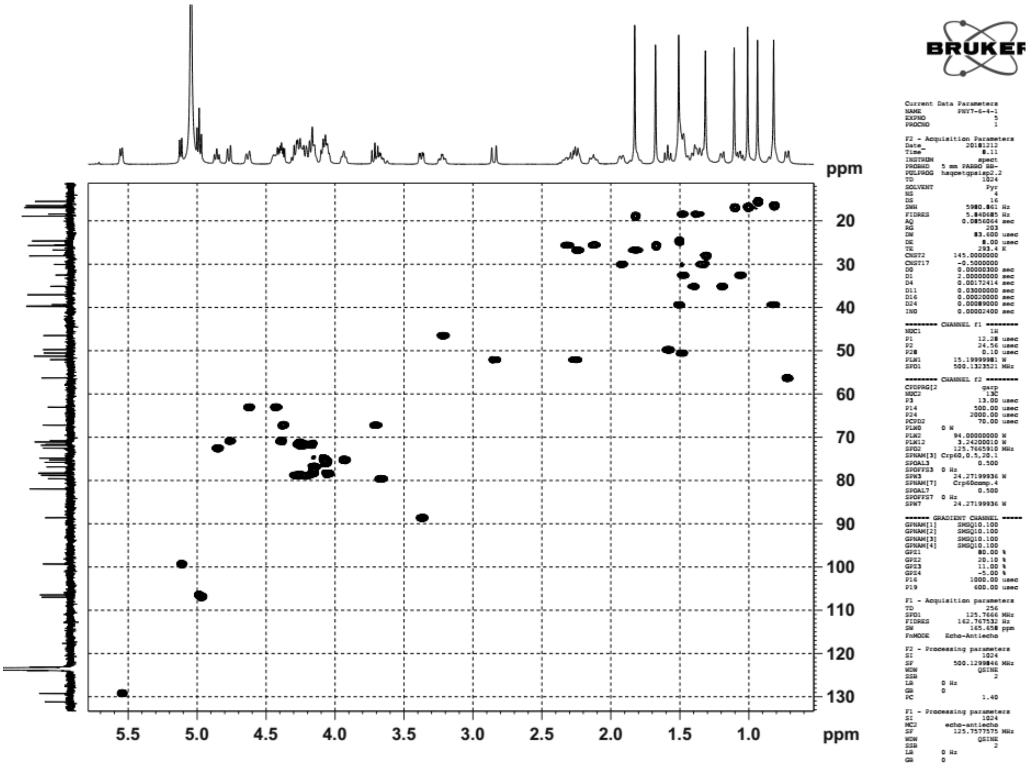

Figure S4 HSQC (C_5D_5N) spectrum of **1**

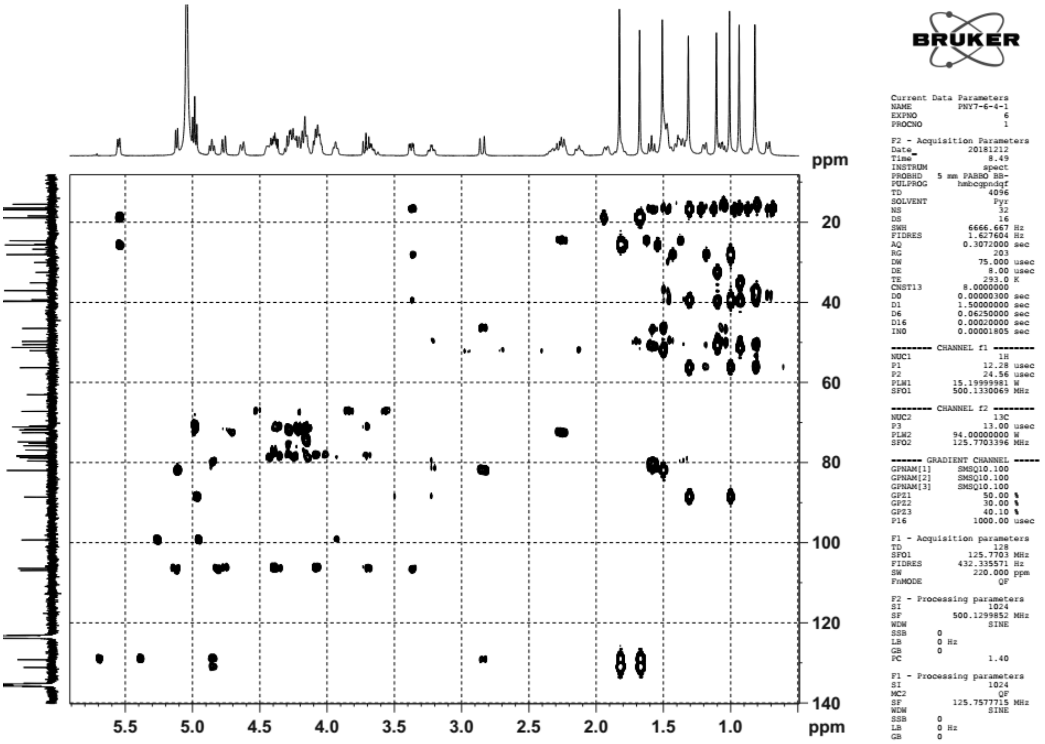

Figure S5 HMBC (C_5D_5N) spectrum of **1**

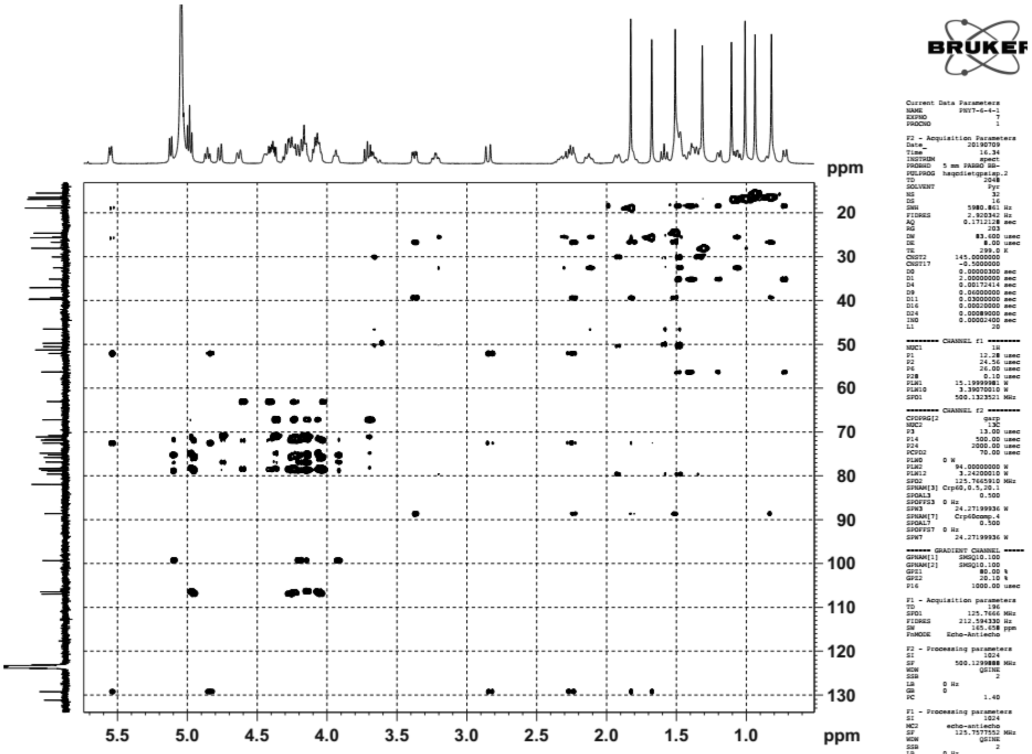

Figure S6 HSQC-TOCSY (C_5D_5N) spectrum of **1**

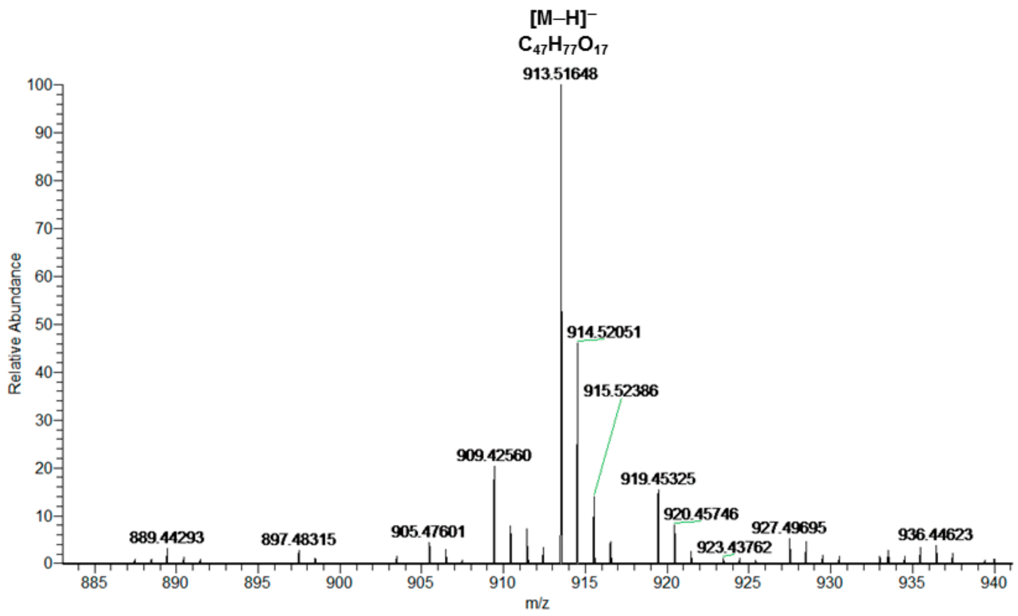

Figure S7 ESI-Q-Orbitrap-MS spectrum of **1**

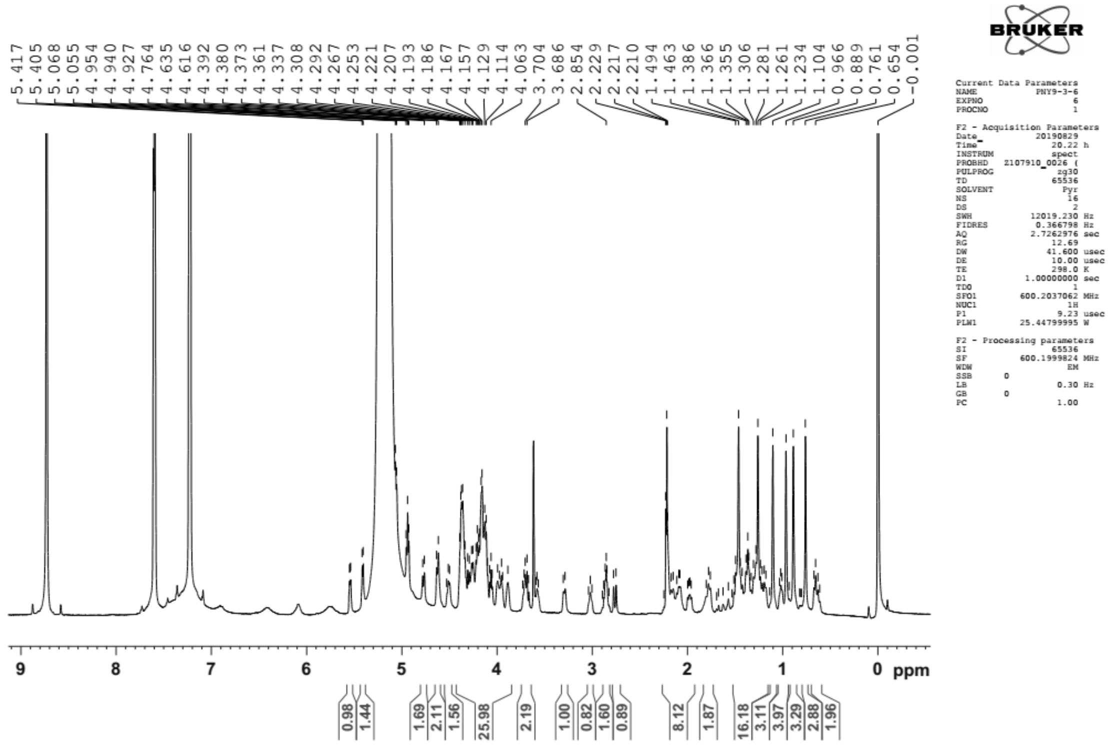

Figure S8 ^{1}H NMR (600 MHz, C_5D_5N) spectrum of **2**

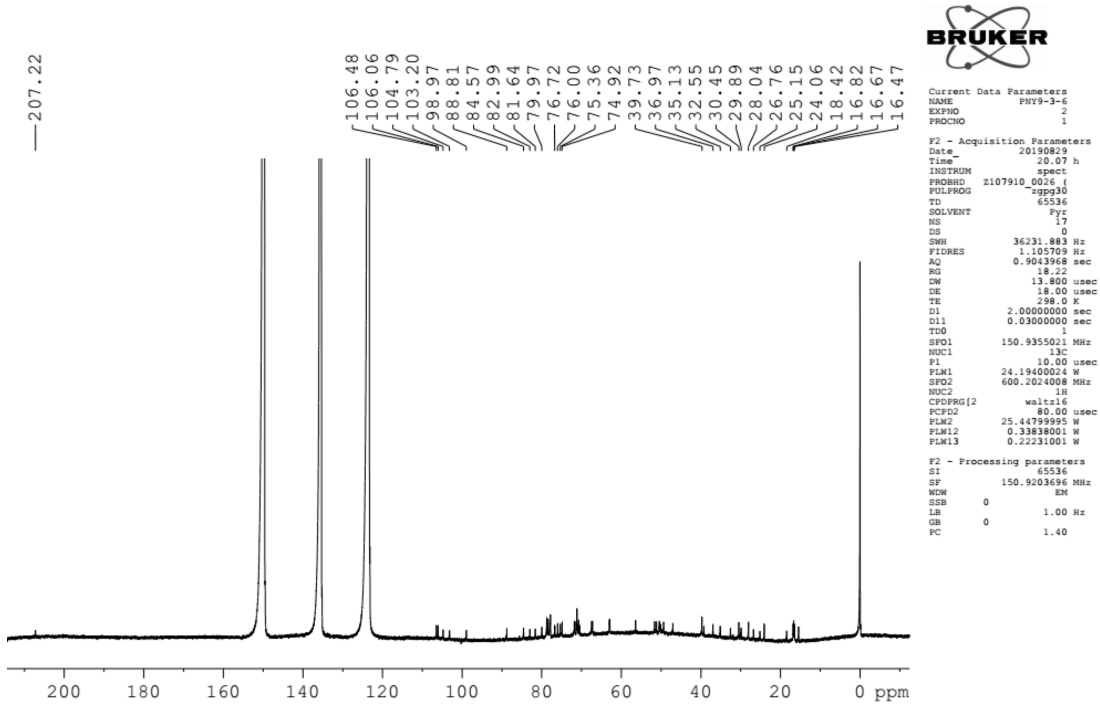

Figure S9 ^{13}C NMR (150 MHz, C_5D_5N) spectrum of **2**

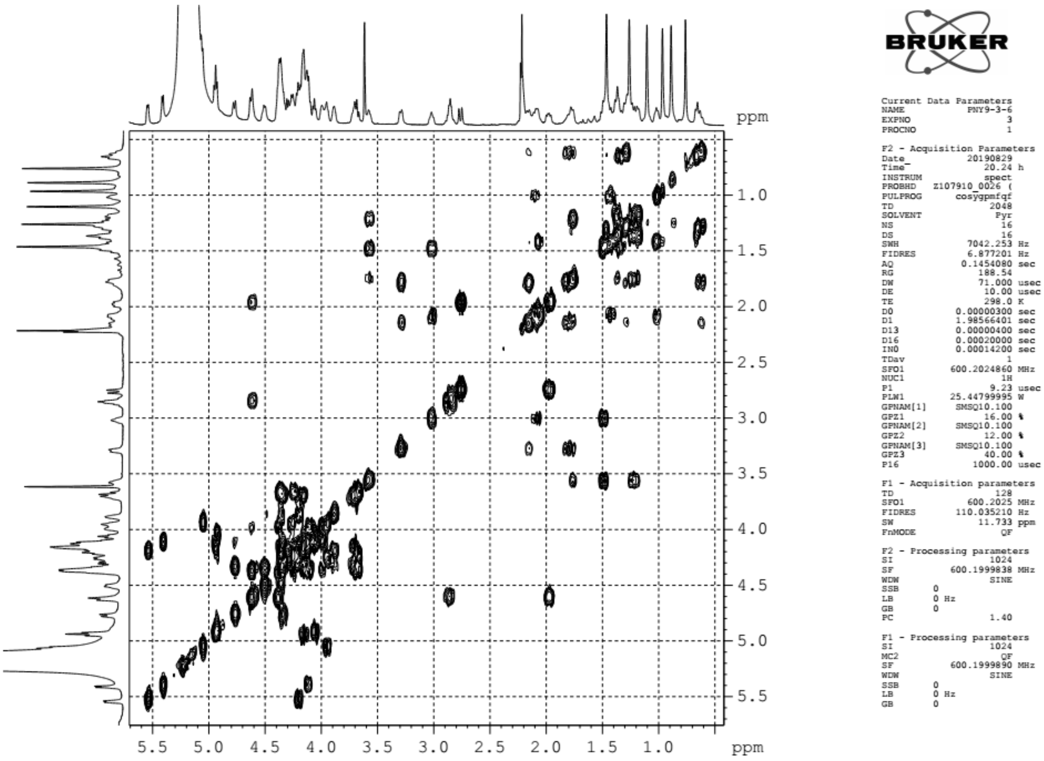

Figure S10 ^{1}H ^{1}H COSY (C_5D_5N) spectrum of **2**

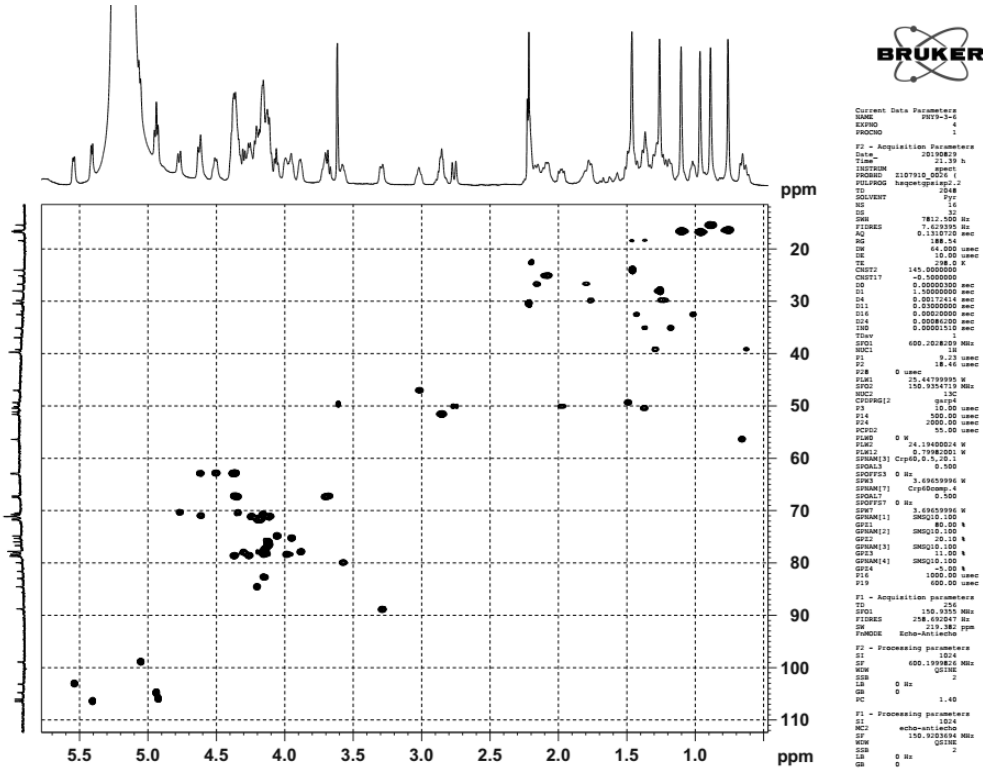

Figure S11 HSQC (C_5D_5N) spectrum of **2**

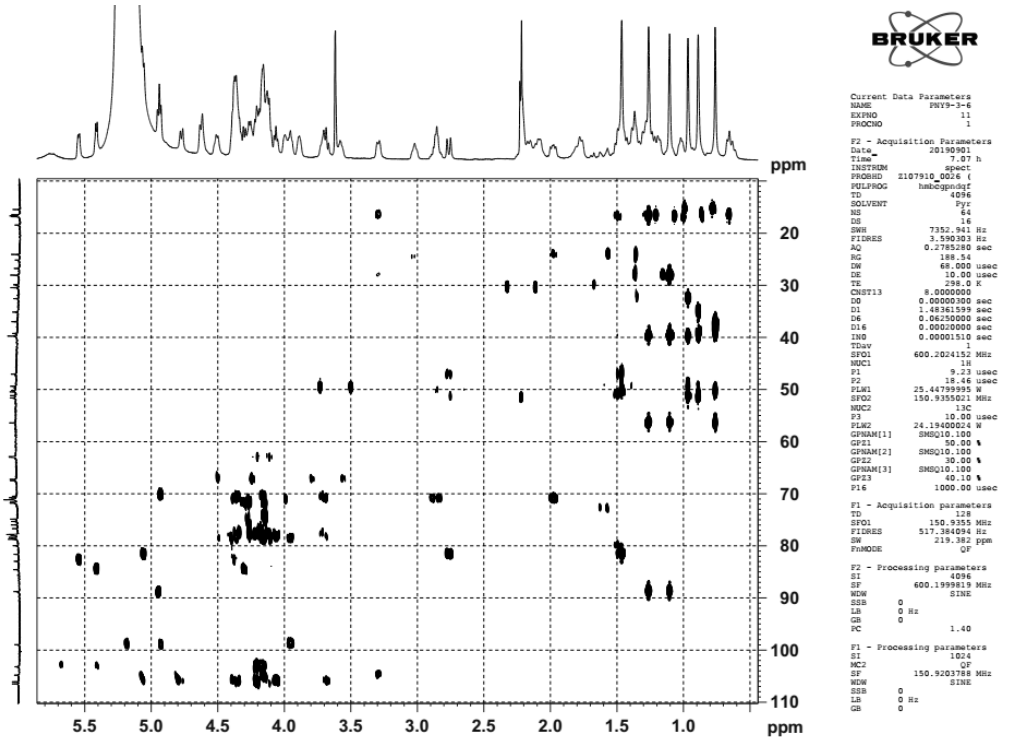

Figure S12 HMBC (C_5D_5N) spectrum of **2**

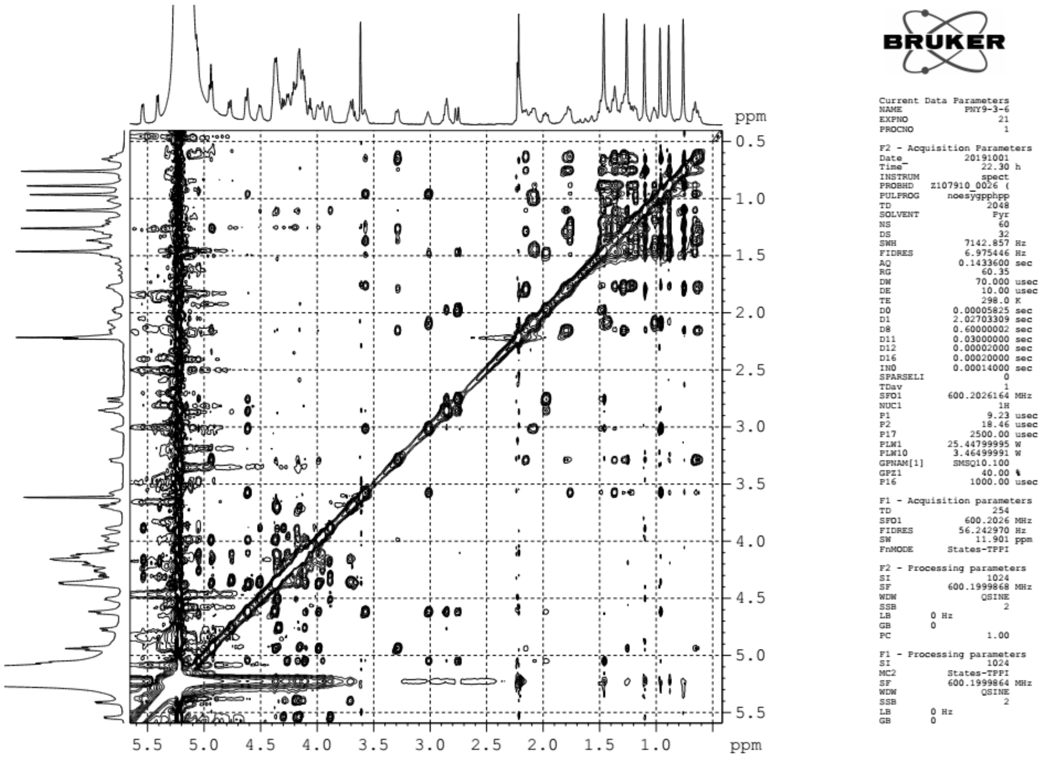

Figure S13 NOSEY (C_5D_5N) spectrum of **2**

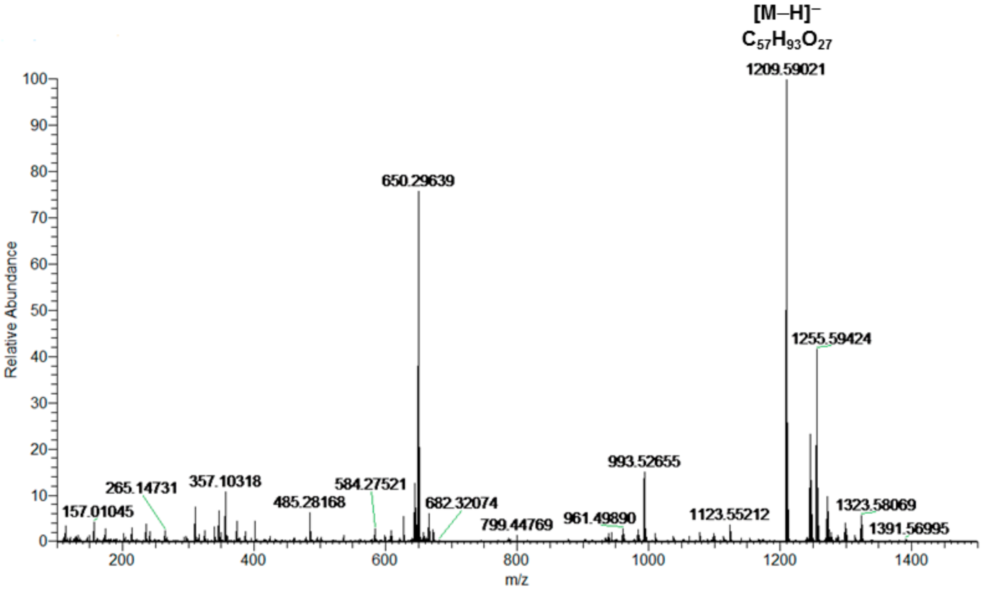

Figure S14 ESI-Q-Orbitrap-MS spectrum of **2**

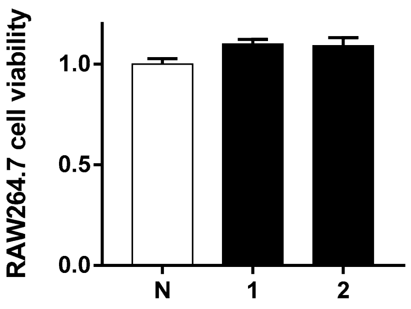

Figure S15 MTT assay of compounds **1** and **2**
at the concentration of 25 μM on RAW 264.7 cells

Cell viability: percentage of normal group (set as 100%). Values represent the mean ± SD of four determinations. n= 4.

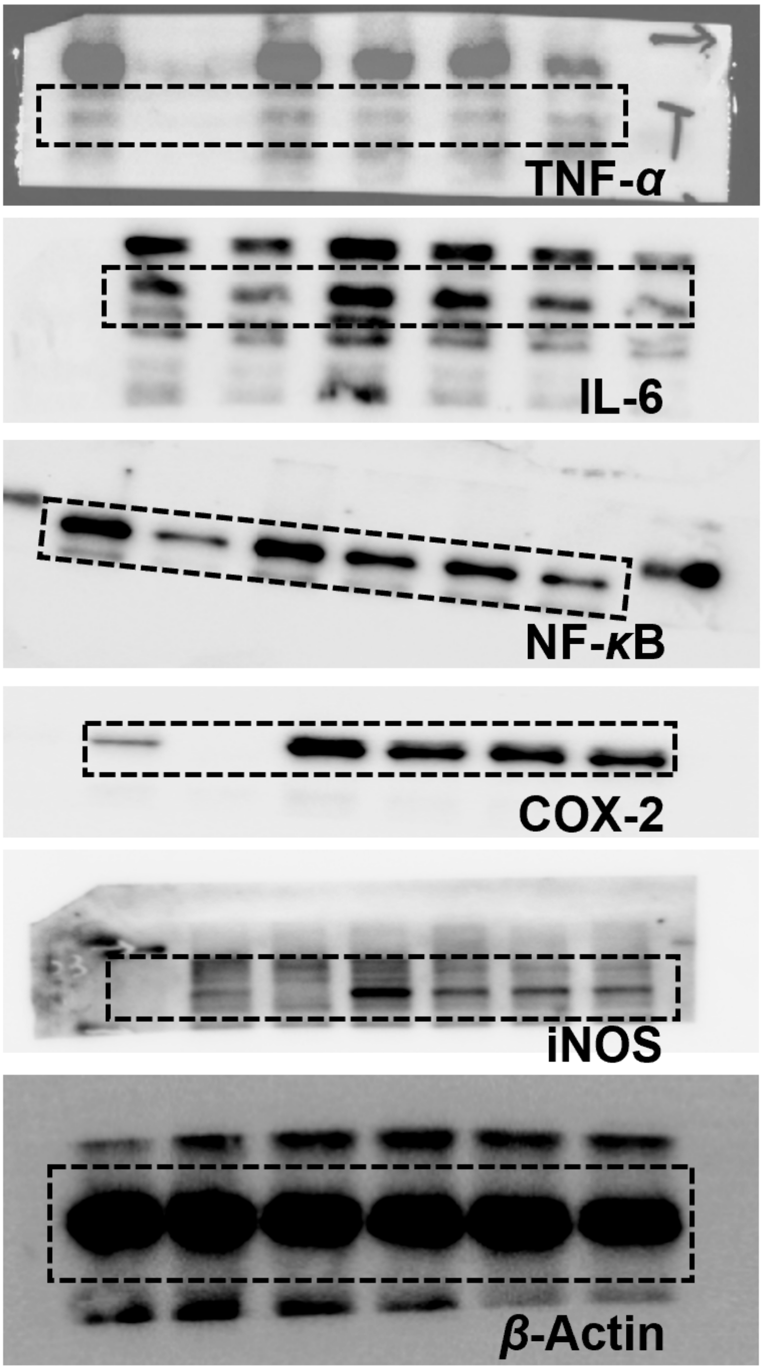

Figure S16 Raw quantification data for figure 3

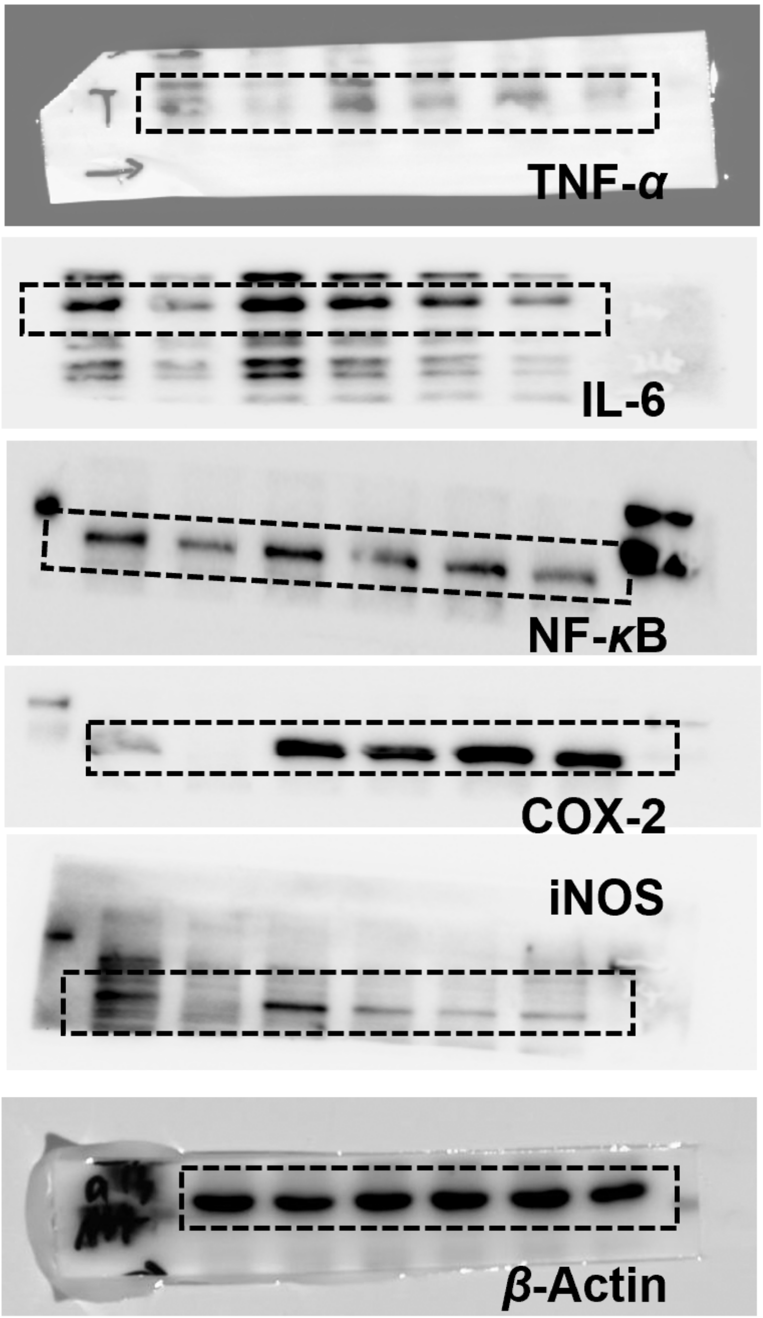

Figure S17 Raw quantification data for figure 4
